# Predicting Soil Salinity with Vis–NIR Spectra after Removing the Effects of Soil Moisture Using External Parameter Orthogonalization

**DOI:** 10.1371/journal.pone.0140688

**Published:** 2015-10-15

**Authors:** Ya Liu, Xianzhang Pan, Changkun Wang, Yanli Li, Rongjie Shi

**Affiliations:** 1 Key Laboratory of Soil Environment and Pollution Remediation, Institute of Soil Science, Chinese Academy of Sciences, Nanjing, 210008, China; 2 University of Chinese Academy of Sciences, Beijing, 100049, China; Old Dominion Univ., UNITED STATES

## Abstract

Robust models for predicting soil salinity that use visible and near-infrared (vis–NIR) reflectance spectroscopy are needed to better quantify soil salinity in agricultural fields. Currently available models are not sufficiently robust for variable soil moisture contents. Thus, we used external parameter orthogonalization (EPO), which effectively projects spectra onto the subspace orthogonal to unwanted variation, to remove the variations caused by an external factor, e.g., the influences of soil moisture on spectral reflectance. In this study, 570 spectra between 380 and 2400 nm were obtained from soils with various soil moisture contents and salt concentrations in the laboratory; 3 soil types × 10 salt concentrations × 19 soil moisture levels were used. To examine the effectiveness of EPO, we compared the partial least squares regression (PLSR) results established from spectra with and without EPO correction. The EPO method effectively removed the effects of moisture, and the accuracy and robustness of the soil salt contents (SSCs) prediction model, which was built using the EPO-corrected spectra under various soil moisture conditions, were significantly improved relative to the spectra without EPO correction. This study contributes to the removal of soil moisture effects from soil salinity estimations when using vis–NIR reflectance spectroscopy and can assist others in quantifying soil salinity in the future.

## Introduction

Soil salinization is a primary ecological degradation process, especially in arid and semi-arid areas [1], that limits water uptake by crops (by reducing the osmotic potential and the total soil water potential) [2], inhibits crop growth and reduces crop yield [1]. Thus, timely detection of the extent and magnitude of soil salinity is important for agriculture practices [3–5].

It is difficult to obtain up-to-date soil salinity information by using conventional techniques to identify and monitor soil salinity because these techniques are time consuming and expensive and require high sampling densities and frequencies [6, 7]. Efforts are being made to obtain more cost-effective methods for mapping soil salinity. During the last two decades, visible and near-infrared (vis—NIR) spectroscopy has been used as a rapid, cost-effective and relatively accurate method for analyzing conventional soil properties [8–10]. Previously, several studies indicated that pure sodium chloride is featureless in vis—NIR regions because salt is not a strong or direct chromophore [11, 12]. However, the presence of salts in soils may result in subtle spectral responses when combined with—OH, which is common in soils [8]. Therefore, some studies have shown that soil salinity can be characterized by soil spectral reflectance or salinity spectral indexes using partial least squares regression, artificial neural network, and stepwise multiple linear regression methods [5,13,14] and can be detected using high-resolution spectroscopy [15–18]. Accordingly, interest in using vis—NIR spectral reflectance as a rapid and effective tool for mapping soil salinity has recently grown.

Several studies have estimated the soil salt contents (SSCs) of air-dried soils with reasonable accuracy using vis—NIR reflectance spectroscopy [13]. However, when vis—NIR is deployed in the field, several external factors, including soil moisture, a major obstacle for vis—NIR field applications, influence the prediction accuracy [19]. Because soil moisture influences the entire optical domain [20], especially the infrared domain [21], it may dramatically degrade the prediction accuracy of several soil properties [5, 22–24]. Therefore, a method for removing the effects of soil moisture from reflectance spectra is urgently needed.

Recently, the external parameter orthogonalization (EPO) algorithm, which was initially designed by Roger et al. [25] to correct for temperature effects when predicting the sugar contents of intact fruits, was successfully applied to remove unwanted disturbances from spectra collected from several fields. Amat-Tose et al. [26] applied EPO to improve the prediction values of two major properties of gasoline, and Hansen et al. [27] used EPO to detect variations in ultrafiltrated milk permeates. Minasny et al. [28] initially applied EPO to eliminate the effects of soil moisture on predicting soil organic matter contents, and Wang et al. [29] later used the EPO algorithm to construct a more robust calibration model for estimating crop residue cover. Both of these researchers attempted to adjust the EPO algorithm for their specific purposes.

This paper investigated an improved method for predicting soil salinity by using vis—NIR spectra after removing the effects of soil moisture by using EPO. The main objectives of this study were (i) to adjust the EPO algorithm according to our specific study objects (soils with a wider range of soil moisture) and verify the effectiveness of the algorithm for removing soil moisture effects from vis—NIR spectra and (ii) to evaluate the performance of EPO corrected spectra for predicting soil salinity by comparing the partial least squares regression (PLSR) prediction results of the EPO corrected and uncorrected spectra.

## Materials and Methods

### Ethics statement

The authors confirm that none of the soil samples were collected from national parks or other protected areas. No specific permissions were required for the soil sampling locations/activities. In addition, the authors confirm that the field studies did not involve endangered or protected species.

### Laboratory experiment design

**Fig 1:**
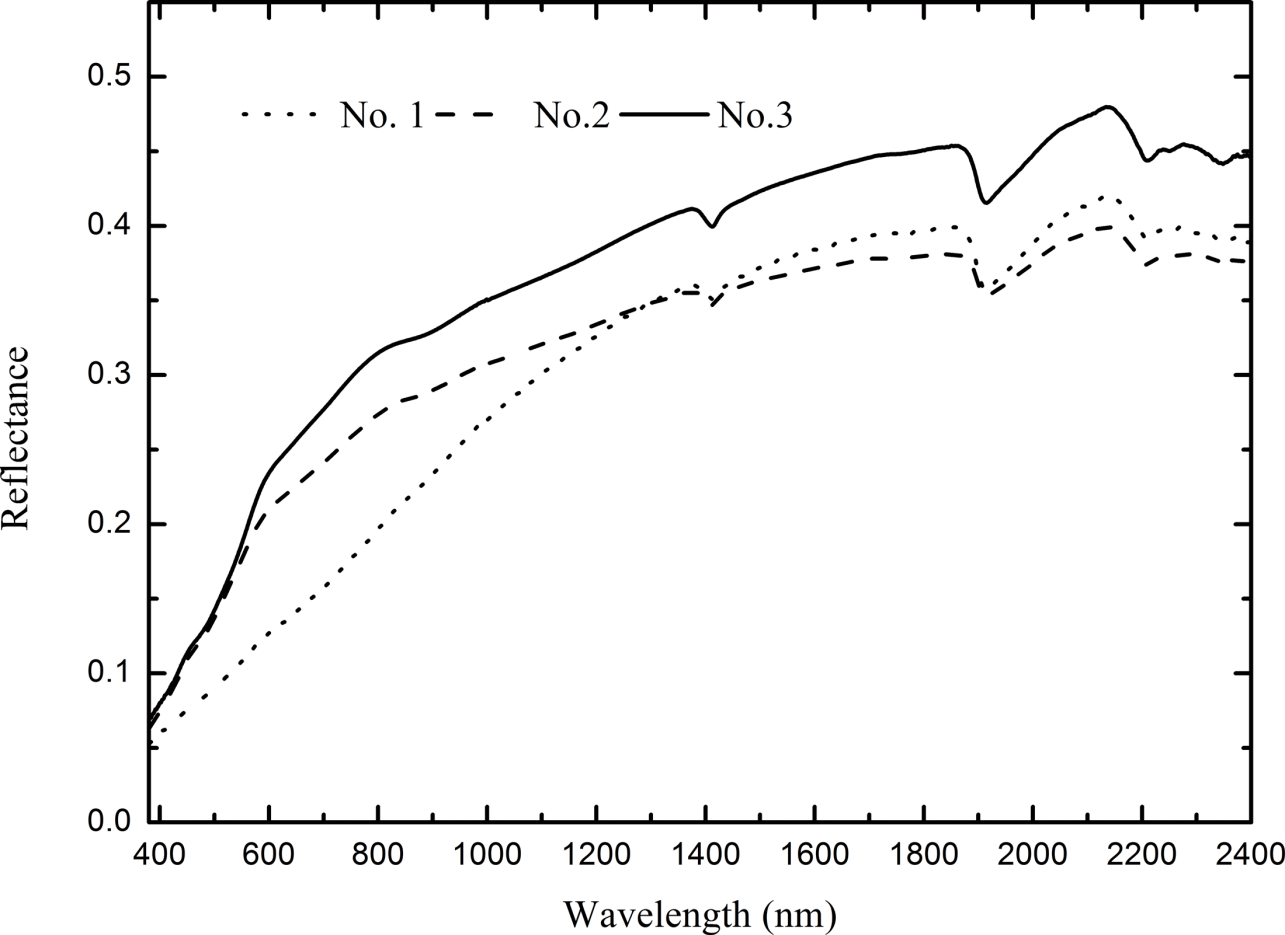
Original reflectance spectra of the three base soils.

Before conducting the laboratory experiment, 102 soil samples were collected from a typical coastal plain area in Dafeng County, Jiangsu Province, China. The soil samples were classified as Solonchaks according to the World Reference Base for Soil Resources [30] and had a silt loam texture according to the USDA texture classification system. The soils developed from similar parent materials (marine sediments) and had slightly different characteristics. However, these samples did not meet our experimental demands because their salt contents covered a narrow range. Therefore, a controlled experiment using three randomly selected soils was designed to acquire artificial soil samples with various salt and moisture contents. The properties of the three base soils are shown in Table 1, and the original spectra of the three base soils are presented in Fig 1.

**Table 1:** Physical-chemical properties of the base soils.

Base soil ID	SOM	Salt contents	Total N	Total P	Total K	pH	Clay	Silt	Sand
	g kg^-1^					%			
No. 1	18.61	0.03	0.96	0.94	17.30	8.3	11.0	70.6	18.3
No. 2	17.85	0.09	1.01	0.68	17.75	8.1	8.4	79.1	12.5
No. 3	13.53	0.04	0.92	0.92	17.40	8.1	7.0	81.5	11.5

Our previous studies revealed that Cl^−^ and Na^+^ were the predominant anions and cations in the sampling zone. We observed a strong correlation between these two ions [31]; thus, a NaCl solution was added to the three base soils to create soil samples with different salt levels. Each of the three base soils was air-dried, passed through a 2-mm sieve, and placed into ten dishes (diameter of 6.5 cm and depth of 2 cm) that contained small holes in the bottom to allow the NaCl solution to enter the soil. After weighing, the 30 dishes were placed in 10 shallow containers with distilled water or salt solutions (0.15, 0.3, 0.6, 0.9, 1.2, 1.6, 2, 3 and 3.5%; w/w) and then removed and drained for 12 h. Next, the dishes were weighed to calculate the salt (g kg^-1^) and moisture (g g^-1^) contents of each soil sample, and the 30 soil samples (3 base soils × 10 salt concentrations) with various salt contents were prepared for evaporation to obtain various soil moisture contents.

The samples were placed in the laboratory to simulate evaporation and were weighed every 12 h, and the soil reflectance spectra were measured for each sample using an ASD spectrometer (Fieldspec 3 Hi-Res, PANalytical, Boulder, CO, USA) with wavelengths of 350 to 2500 nm and a spectral resampling interval of 1 nm. Soil samples were illuminated using two 50-W quartz-halogen lamps, which were placed to collect light beams that were 45° from vertical using an 8° field of view sensor that was perpendicular to the sample at a distance of 25 cm. A white Spectralon panel (25 by 25 cm; Labsphere, Inc., North Sutton, NH, USA) was used to obtain the reflectance factor and was calculated as the sample reading divided by the reference panel reading. Five reflectance spectra were measured in four directions, each by successively rotating the sample by 90° between readings. The 20 spectra were averaged to minimize instrument noise, and all spectral measurements were performed in a dark room to reduce the effects of light scattering.

When the weight and spectral reflectance changes of the samples were no longer dramatic, the sample observation was completed. The observation period lasted approximately 10 days, and 570 spectra were obtained from the 30 soil samples at 19 soil moisture content levels (S1 File).

### External parameter orthogonalization

To improve the calibration performance, an EPO algorithm was used to remove unwanted disturbance effects and reduce the effects of soil moisture on spectral reflectance. The EPO algorithm was used to eliminate the influences of clearly identified factors by using a characterized spectrum [32]. This algorithm detects areas in the spectra that are affected by external factors and projects the spectra onto the subspace orthogonal to this variation, which allows the undesired variations to be successfully eliminated [28]. The theory of the EPO algorithm [25, 29] is demonstrated below (capital bold characters will be used for matrices (e.g., **X**), lowercase bold italic characters will be used for column vectors, and ***x***_i_ will denote the *i*th row of **X**).

If **X** (size *m* × *p*) is defined as the matrix of the measured spectra (*m*) in the dimension space affected by the external parameter (*p*), the spectra matrix (**X)** can be written as follows:

X=XP+XQ+R (1)

where **P** is the projection matrix (size *p* × *p*) of the portion of the spectra that is influenced by the variable of interest, **Q** is the projection matrix (size *p* × *p*) of the portion of the spectra affected by the external parameters; and **R** is the residual matrix (size *m* × *p*). The objective of EPO is to separate the useful portion (**X** ^*^ = **XP**) from the undesirable portion (**X** ^**#**^ = **XQ**).

The estimated **Q** ($\hat{\text{Q}}$) can be calculated as follows:

$$\hat{\text{Q}}=\hat{\text{G}}{\hat{\text{G}}}^{\text{T}}$$ (2)

where $\hat{\text{G}}$ (size *p* × *k*, *k* < *p*) is the standard orthogonal matrix of **X** ^**#**^. Thus, an estimation $\hat{\text{P}}$ of **P** can be obtained as follows:

$$\hat{\text{P}}=\text{I}-\hat{\text{G}}{\hat{\text{G}}}^{\text{T}}$$ (3)

Therefore, the estimated **X** ^*^ (${\hat{\text{X}}}^{*}$) can be calculated as follows:

X^=*XP^ (4)

The matrix of $\hat{\text{G}}$ can be obtained by computing the PCA of **D** (size *n* × *p*), which is defined as a difference matrix and includes adequate variations (here, *n* levels) that are induced by an external parameter [25]. In addition, **D** is calculated as follows:

$$d_{h}=x_{h}-x_{1}$$ (5)

where ***x***_*h*_ is the mean spectrum among the spectra that are influenced by the *h*th variation in the external parameter, and the value of *h* is between 1 and *n*; thus, ***d***_1_ = 0.

Because the first *k* principal components normally represent the most information for **D**, $\hat{\text{G}}$ can be built using the first *k* eigenvectors. If *k* = *p*, the EPO algorithm will remove all of the information regarding the response of the variable of interest. Thus, the largest value of *k* was *p*-1, and for each value of *k* (from 1 up to *p*-1), $\hat{\text{G}}$ can be obtained. Next, $\hat{\text{Q}}$, $\hat{\text{P}}$ and ${\hat{\text{X}}}^{*}$ can be calculated, and the optimal value of *k* can be determined from the coefficient of determination (*R* ^2^_cv_) and the root mean square error (RMSE_cv_) values in the cross validation step.

In this study, we identified the responses of various SSCs and eliminated the influences of soil moisture on spectral reflectance. In addition, all of the spectra before the EPO process were subjected to standard normal variate (SNV) pre-processing [33] to reduce the multiplicative interferences of scatter and particle size [26,28,34] by using Eq (6).

$${\text{X}}_{\text{ij}}(\text{SNV})=\frac{{\text{X}}_{\text{ij}}-{\bar{\text{x}}}_{\text{i}}}{{\text{SDev}}_{\text{i}}}$$ (6)

where *i* is the spectrum counter, *j* is the reflectance value counter of the *i*th spectrum, X_ij_(SNV) is the corrected reflectance value, X_ij_ is the measured reflectance value, ${\bar{\text{x}}}_{\text{i}}$ is the mean uncorrected value of the *i*th spectrum, and SDev_i_ is the standard deviation of the reflectance values of the *i*th spectrum.

The SNV spectra of the three base soils were defined as **X**_**all**_, for which a global **P**_**all**_ value was calculated from the global **D**_**all**_ value. Then, **P**_**all**_ was used to correct **X**_**all**_, and the corrected spectra were used to form dataset B.

The EPO process was performed using the four steps outlined below [25, 26, 28, 29].

1. The mean spectrum corresponding to the *m* SNV spectra was calculated for *n* different soil moisture contents by dividing all of the soil moisture contents into *n* levels at 0.03 g g^-1^ intervals (*n* = 17 according to our experiment in which the soil moisture contents spanned from 0.02 to 0.51 g g^-1^).
2. The difference spectra matrix, **D** (size *n* × *p*), was calculated using Eq (5), where *p* is the number of wavelengths and ***x***_**1**_ is the mean of the spectra collected from soil samples with moisture contents less than 0.03 g g^-1^.
3. PCA was performed on **D** to obtain a base **G** (size *p* × *k*), which is the *k* first component with *k* < *p*. The optimal value of *k* can be determined from the coefficient of determination (*R* ^2^_cv_) and the root mean square error (RMSE_cv_) in the cross validation step [25].
4. The projection matrix **P** (size *p* × *p*) was calculated using Eq (3). Next, the transformed spectra matrix was calculated using Eq (4), where **X** (size *m* × *p*) is the spectra matrix built with *m* spectra after SNV pre-processing.

The EPO algorithm was performed using Matlab 7.9.0 (MathWorks Inc., Natick, MA, USA).

### Partial least squares regression

Partial least squares regression (PLSR) was applied to establish a correlation between the soil spectral values (independent variable) and the SSCs (dependent variable). Because the reflectance data in the extreme wavelength ranges of 350–379 nm and 2401–2500 nm were significantly affected by noise, the reflectance data between 380–2400 nm were used for the PLSR analysis. Two datasets were used for the PLSR analysis, the SNV reflectance data without EPO correction were identified as dataset A, and the spectra in dataset A after EPO correction were identified as dataset B. Both of the datasets contained 570 (3 base soils × 10 salt concentrations × 19 soil moisture levels) sets of data. Each dataset was divided into two subsets, with one-third of the dataset (selected every third set of data) forming the prediction set and the remaining two-thirds of the dataset forming the calibration set. Four subsets were sampled and operationally defined as subsets A_c_ (N = 380), A_p_ (N = 190), B_c_ (N = 380) and B_p_ (N = 190). Normally, cross validation can be used to test how well a model fits a hypothetical validation set when an additional validation dataset is not available (limited by the number of samples) [35]. In our study, enough samples were collected to build a validation dataset. Thus, cross validation performed in calibration was only used to choose the optimal PLSR factors (*c*) and EPO dimensions (*k*) by comparing the *R* ^2^_cv_ and RMSE_cv_ values. Validation of the optimal calibration model obtained by cross validation was performed using the prediction data sets (subsets A_p_ and B_p_).

For dataset A, the *c* parameter must be optimized, which corresponds to the number of PLSR factors (latent variables or orthogonal factors). Thus, we selected the optimal *c* value by choosing the lowest RMSE_cv_ and *R* ^2^_cv_ values of the calibration models with different *c* values. When the number of PLSR factors that resulted in the lowest RMSE_cv_ values did not correspond with the number of PLSR factors that resulted in the largest *R* ^2^_cv_ values, *R* ^2^_cv_ was considered more important than RMSE_cv_. We calculated the RMSE_cv_ and *R* ^2^_cv_ values using 20 more PLSR factors, which indicated that the RMSE_cv_ and *R* ^2^_cv_ values did not change after 20 PLSR factors. Therefore, the use of additional PLSR factors was meaningless and ineffective. Consequently, we used a maximum *c* value of 20 in this study to show the RMSE_cv_ and *R* ^2^_cv_ results. For a range of PLSR factors (*c* values of 1 to 20), we derived different PLSR calibration models by using the subset A_c_ (calibration dataset of SNV spectra; N = 380). Next, we calculated the RMSE_cv_ and *R* ^2^_cv_ values of the calibration models for different *c* values and selected the optimal *c* value with the lowest RMSE_cv_ and highest *R* ^2^_cv_ values.

In addition to the *c* value, the optimal value of *k* must be determined for dataset B, which is the number of EPO dimensions. For each *k* value between 1 and 10, we calculated the projection matrix **P** and then corrected the dataset using **P**. The PLSR model was established for each value of *c* (from 1 to 20) for each dataset formed by the corrected spectra using each value of *k*. Thus, there were *k* × *c* (the combination of *k* and *c*) PLSR models in the process, and the optimal numbers for *k* and *c* were determined by using the lowest RMSE_cv_ and highest *R* ^2^_cv_ values.

The PLSR model was implemented using the Unscrambler 9.7 software (Computer Aided Modeling, Trondheim, Norway).

### Accuracy assessment

Various parameters were calculated to determine the accuracies of the models, including the coefficient of determination in the cross validation (*R* ^2^_cv_), the coefficient of determination in the prediction (*R* ^2^_p_), the root mean square error of cross validation (RMSE_cv_), the root mean square error of prediction (RMSE_p_), and the ratio of the standard deviation to the root mean square error of prediction (RPD).

$$\text{RPD}=\text{SD}/{\text{RMSE}}_{\text{p}}$$ (7)

where SD is the standard deviation of the measured values of the prediction samples.

## Results

### Soil moisture effects before and after EPO

**Fig 2:**
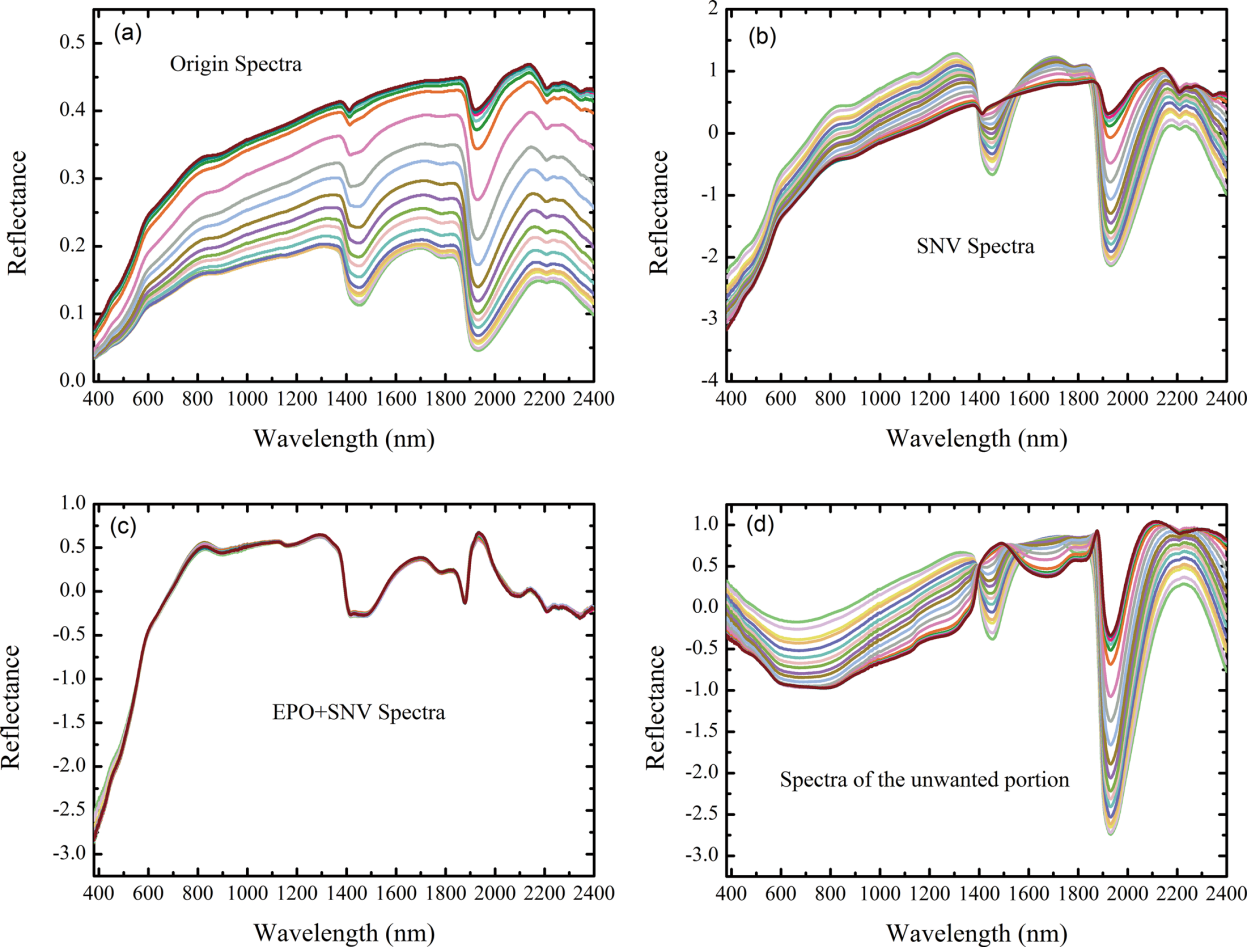
(a) The original reflectance spectra, (b) SNV spectra, (c) EPO spectra and (d) the spectra of the unwanted portion of one sample with the same soil salt content and different soil moisture contents (g g^-1^).

As expected, the original spectra from the soils with the same salt contents were different under different moisture conditions (Fig 2a). The shapes of the spectra were similar, with obvious absorption features near 1400, 1900, and 2200 nm. These features are mainly associated with—OH (e.g., the—OH features of free water occur at 1400 and 1900 nm and lattice—OH features occur at 1400 and 2200 nm [36]). The reflectance value across the entire wavelength domain decreased as the soil moisture content increased; thus, the soil moisture content had a large influence on reflectance, which masked the subtle responses of various salt contents on reflectance and should not be ignored.

After EPO correction, which transformed the spectra into a space that was nearly unaffected by moisture content, the condition remarkably improved. SNV minimized the differences between the original spectra and the spectra obtained at various soil moisture contents (Fig 2a vs. 2b). However, the shapes of the SNV spectra were different, especially near the water absorption bands of approximately 1400 and 1900, and 2200 nm, which were related to clay minerals. The SNV transformation that was performed on the individual spectrum was intended to reduce spectral noise and eliminate background effects. Obviously, the SNV transformation could not correct the spectra for soil moisture effects; therefore, the soil spectra varied for different soil moisture conditions.

However, after EPO correction, the spectra under various soil moisture conditions were identical, and the differences among the water absorption bands were not obvious (Fig 2c). The spectra representing the unwanted portion are shown in Fig 2d, and the spectra from the soil samples with different moisture contents were different at all wavelengths, especially near the major absorption wavelengths of 1400, 1900 and 2200 nm. Thus, the EPO algorithm successfully eliminated the effects of soil moisture from the spectra.

### Prediction of SSCs using the PLSR method

The EPO correction resulted in an improved spectral response to changes in salt contents once the influence of soil moisture was removed. Therefore, we evaluated the performance of EPO corrections for predicting SSCs by PLSR.

**Fig 3:**
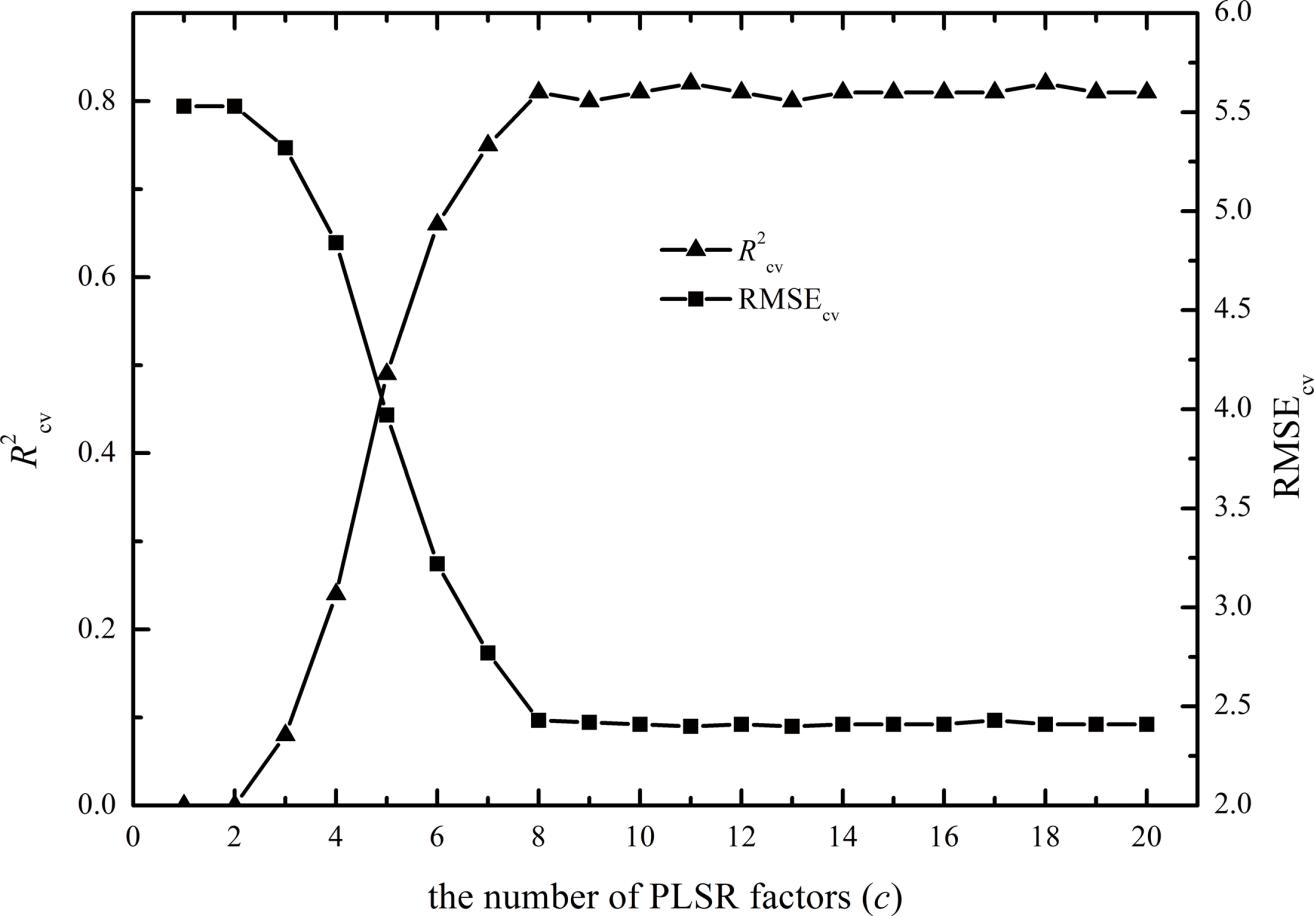
Parameters of the PLSR calibration model.

For dataset A, the RMSE_cv_ and *R* ^2^_cv_ values did not obviously change when the PLSR factor was greater than 8 (Fig 3). Therefore, we selected *c* = 8.

**Fig 4:**
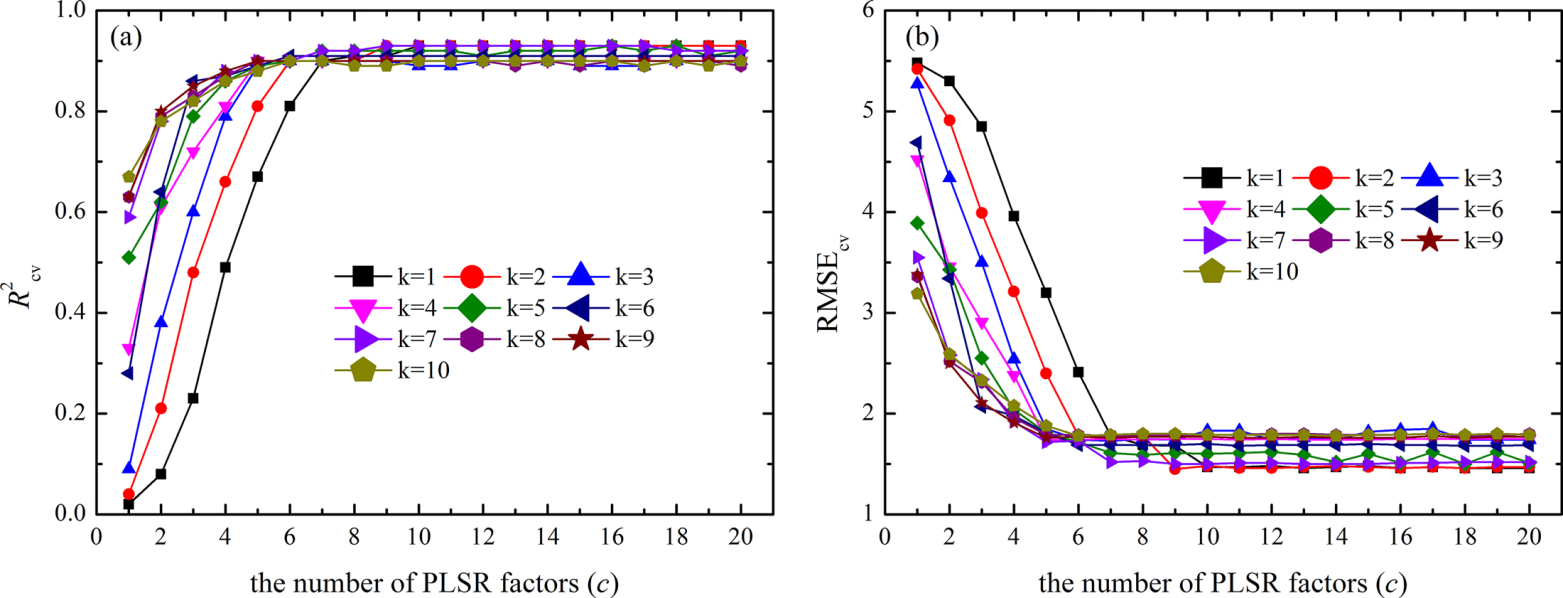
(a) *R* ^2^_cv_ and (b) RMSE_cv_ values of SSCs in dataset B using the corrected spectra with different numbers for the EPO dimension (*k*, denoted in the legend) and PLSR factor (*c*, denoted on the x- axis).

For dataset B, the highest *R* ^2^_cv_ (0.93) and lowest RMSE_cv_ (1.45 g kg^-1^) values were obtained when using an EPO dimension of 2, and the *R* ^2^_cv_ and RMSE_cv_ values did not obviously change when the PLSR factor was greater than 9 (Fig 4). Therefore, we selected *k* = 2 and *c* = 9.

**Fig 5:**
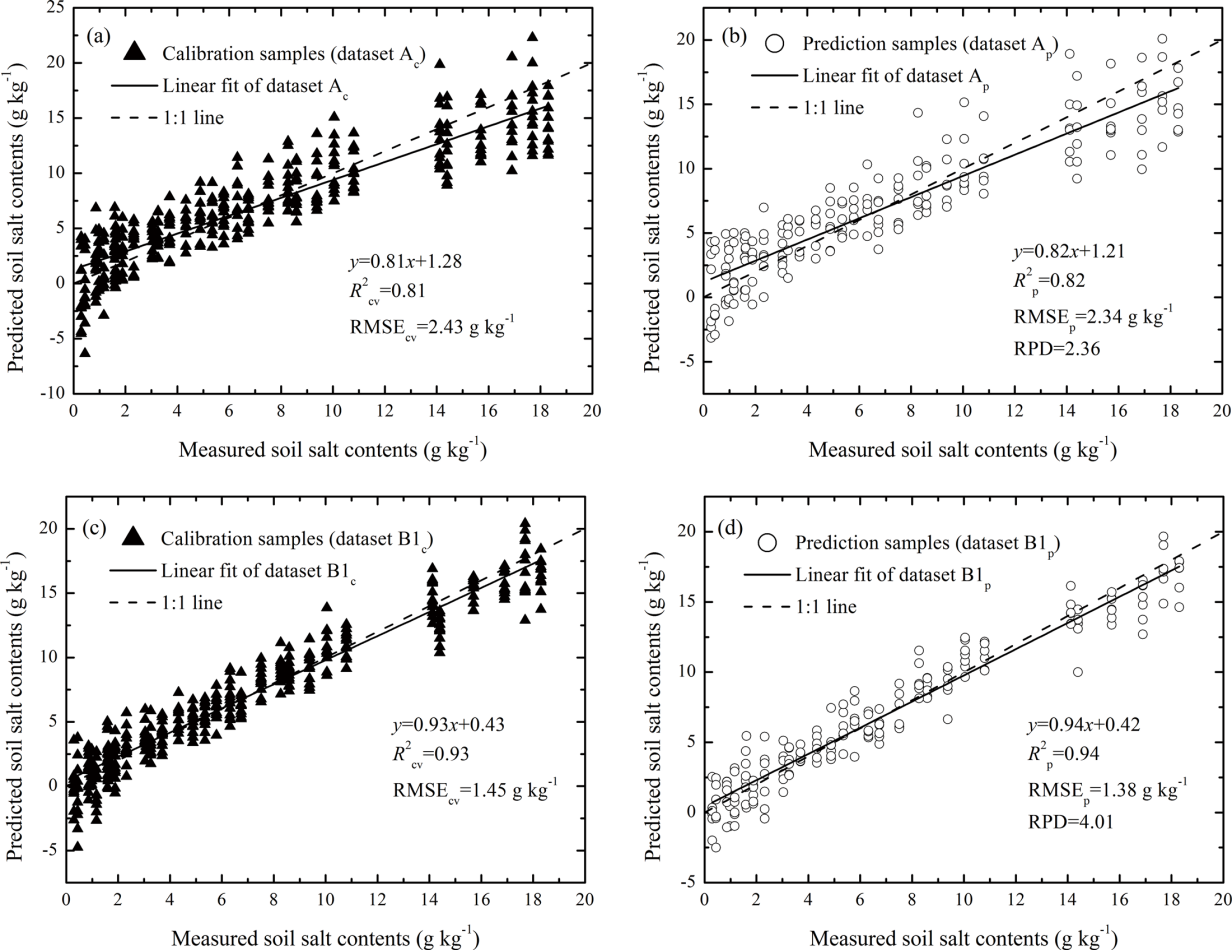
Scatter plots of the measured vs. predicted soil salt contents (SSCs) that were derived from the PLSR analysis of soil spectra based on subsets (a) A_c_, (b) A_p_, (c) B_c_, and (d) B_p_.

The results of datasets A and B using optimum parameters are presented in Fig 5, and the PLSR calibration of subset A_c_ (8 PLSR factors) presented the following results: *R* ^2^_cv_ = 0.81 and RMSE_cv_ = 2.43 g kg^−1^ (Fig 5a). Using the model calibrated by subset A_c_, the SSCs were predicted using subset A_p_ with the following values: *R* ^2^_p_ = 0.82, RMSE_p_ = 2.34 g kg^-1^ and RPD = 2.36. In this case, the fitted linear regression line deviated from the 1:1 line (Fig 5b). However, for dataset B, the calibration and prediction results both improved relative to dataset A. The relationships between the measured and predicted SSCs from calibrating and predicting dataset B are shown in Fig 5c and 5d, respectively. The *R* ^2^_p_ values were > 0.90, and the RMSE_p_ values were < 1.50 g kg^-1^. The slope of the fitted linear regression equation of the prediction dataset was nearly 1 with RPD = 4.01 (Fig 5d).

These results suggested that the prediction accuracy was greatly improved, with high *R* ^2^ and RPD values and low RMSE values when the influences of soil moisture on reflectance were removed using EPO relative to uncorrected spectra under various soil moisture conditions.

## Discussion

In this study, soil spectra were obtained at various soil salt and moisture contents. We observed that soil moisture could shape reflectance spectra and mask the subtle effects of SSCs on reflectance spectra. The spectra with the same SSCs were considerably different among the different moisture contents without EPO correction (Fig 2a and 2b); however, the spectra were similar to one another after EPO (Fig 2c), which suggested that the moisture effects were effectively eliminated. The prediction accuracies of the models built on the EPO-corrected spectra were considerably greater than those of the models built on uncorrected spectra, with higher *R* ^2^ and RPD values and lower RMSE values. The better performance of the model built on EPO-corrected spectra mainly occurred because the effects of soil moisture on reflectance were largely removed by the EPO projection.

Many studies have successfully applied the EPO method to remove the effects of external factors from spectral reflectance [25–29]. Compared with the study conducted by Minasny et al. [28], this study provides a better method for establishing the difference matrix **D**, which is important for the EPO process and the experimental design. First, in contrast with our method of using averaged moisture contents from 17 soil samples (at 0.03 g kg^-1^ intervals) to establish **D**, Minasny et al. [28] used rewetted samples from the first and third days. The method used in our study is similar to the method provided by Roger et al. [25], which has been successfully used to remove external parameter effects. Second, the study of Minasny et al. [28] involved rewetted samples with averaged moisture contents of 0 to 18% (gravimetric). Our study considered a wider range of moisture contents (from 2% to 51%) that were closer to the actual field conditions and we used a different rewetting method in which water was added to the bottom of the dish rather than to the soil surface. This method allowed the samples to soak up the water, which resulted in more uniform moisture conditions in each sample and a wider range of soil moisture contents (from air dry to field moisture capacity). The good prediction results (*R* ^2^_p_ > 0.90 and RMSE_p_ < 1.50 g kg^-1^) after the EPO correction indicated that our method for establishing **D** was appropriate in the studied area for eliminating soil moisture effects when predicting SSCs. The results of our study verified that the EPO correction could be applied to soils with all levels of moisture without sacrificing robustness.

The fitted numbers of EPO dimensions (*k*) and PLSR factors (*c*) are important because they significantly affect prediction accuracy and efficiency (Figs 3 and 4); thus, it is important to select optimal numbers for the two parameters. Selecting optimal *c* and *k* values with the highest *R* ^2^ and lowest RMSE values is the most common method for determining these optimal parameters and was used in this study [25, 28, 29].

Because the difference matrix, **D**, can greatly affect estimations of $\hat{\text{G}}$, $\hat{\text{Q}}$ and $\hat{\text{P}}$, the methods and samples used to construct **D** are both important. The **D** matrix was defined as a difference matrix and should be built with spectra that show sufficient variation in their external parameters. Thus, the projection matrix, **P**, can be effective. In this study, all three base soils were used to establish **D,** which contained both soil moisture content variations and soil property variations. Thus, the **P** matrix was effective for all of the soil samples. In addition, the **P** matrix derived from each of the three base soils was used to correct all of the soil samples and yielded satisfactory results. These results indicated that the **P** matrix from a small dataset could be applied to a large dataset, which will be verified in our future work.

Because our study area was not very large and was located on the coastal plain, the 102 soil samples collected from the field all had the same texture according to the WRB and USDA texture classification schemes. Thus, the variations in the three base soils used in this study were limited, it remains unclear whether EPO can be used in areas that are more complex, and the number of samples required to constitute **D** remains unknown. We are looking forward to solving these unknown factors in our ongoing work.

Because the dominant soluble salt in our study area was sodium chloride (NaCl), our study focused on soils rich in NaCl and did not consider the effects of salt type on the robustness of the EPO model. This effect must be investigated in other areas to determine whether this method is effective for soils containing other types of salts, such as soda, chloride-soda, sulfate-soda and other anion groups. Furthermore, the model we provide was based on controlled laboratory experiments and was not validated for field applications. Field measurements must consider naturally varying salt and moisture conditions and other soil properties (e.g., soil texture) that are different from the conditions used in this study. Thus, additional field investigations should be conducted.

The data processing approach used in this study offers a rapid and inexpensive method for quantifying the spatial variability of soil salinity for agricultural purposes, which can provide appropriate information for managing agricultural inputs, such as tillage, water, fertilizer, and seeds, according to the magnitude of salinization. Because soil moisture is highly variable and difficult to control in the field, we hypothesize that the method proposed in this study will facilitate the acquisition of soil properties from field spectral reflectance with improved efficiency and accuracy at a reduced cost. Thus, the rapid and inexpensive collection of precise, quantitative, fine resolution data using proximal remote sensing will be much easier.

## Conclusions

Soil moisture significantly influences the reflectance of saline soil and reduces the accuracy of soil salinity estimations when using vis—NIR techniques. The EPO method provides an effective method for removing the effects of soil moisture when estimating SSCs and improves the prediction accuracy of SSCs relative to spectra without EPO correction. This approach may facilitate a field method for rapidly measuring SSCs; thus, we expect that this method will be widely applied for large-scale SSCs mapping.

## Supporting Information

S1 File Vis-NIR spectra of 570 samples. To every tenth wavelength was retained to reduce the size of the file.
(XLSX)
